# Analysis of 25 Years of Polar Motion Derived from the DORIS Space Geodetic Technique Using FFT and SSA Methods

**DOI:** 10.3390/s20102823

**Published:** 2020-05-16

**Authors:** Qiaoli Kong, Linggang Zhang, Litao Han, Jinyun Guo, Dezhi Zhang, Wenhao Fang

**Affiliations:** 1 College of Geodesy and Geomatics, Shandong University of Science and Technology, Qingdao 266590, China; kqlabc3334@163.com (Q.K.); 17852152715@139.com (L.Z.); spacegsdust@163.com (J.G.); zhangdezhi_fd@foxmail.com (D.Z.); 15192700186@139.com (W.F.); 2 State Key Laboratory of Mining Disaster Prevention and Control Co-Founded by Shandong Province and Ministry of Science and Technology, Shandong University of Science and Technology, Qingdao 266590, China

**Keywords:** polar motion, DORIS, FFT, SSA

## Abstract

Polar motion (PM) has a close relation to the Earth’s structure and composition, seasonal changes of the atmosphere and oceans, storage of waters, etc. As one of the four major space geodetic techniques, doppler orbitography and radiopositioning integrated by satellite (DORIS) is a mature technique that can monitor PM through precise ground station positioning. There are few articles that have analyzed the PM series derived by the DORIS solution in detail. The aim of this research was to assess the PM time-series based on the DORIS solution, to better capture the time-series. In this paper, Fourier fast transform (FFT) and singular spectrum analysis (SSA) were applied to analyze the 25 years of PM time-series solved by DORIS observation from January 1993 to January 2018, then accurately separate the trend terms and periodic signals, and finally precisely reconstruct the main components. To evaluate the PM time-series derived from DORIS, they were compared with those obtained from EOP 14 C04 (IAU2000). The results showed that the RMSs of the differences in PM between them were 1.594 mas and 1.465 mas in the X and Y directions, respectively. Spectrum analysis using FFT showed that the period of annual wobble was 0.998 years and that of the Chandler wobble was 1.181 years. During the SSA process, after singular value decomposition (SVD), the time-series was reconstructed using the eigenvalues and corresponding eigenvectors, and the results indicated that the trend term, annual wobble, and Chandler wobble components were accurately decomposed and reconstructed, and the component reconstruction results had a precision of 3.858 and 2.387 mas in the X and Y directions, respectively. In addition, the tests also gave reasonable explanations of the phenomena of peaks of differences between the PM parameters derived from DORIS and EOP 14 C04, trend terms, the Chandler wobble, and other signals detected by the SSA and FFT. This research will help the assessment and explanation of PM time-series and will offer a good method for the prediction of pole shifts.

## 1. Introduction

Polar motion (PM) describes the motion of the rotation axis of the Earth, relative to the crust, and is closely related to the Earth’s structure and composition. Many geophysical changes of the Earth cause movements of the pole, such as melting ice sheets, the global water cycle, sea level variations, postglacial mass readjustments, seasonal changes of the atmosphere, and other factors [1]. The study of PM can provide valuable information for studying many geophysical and meteorological phenomena [2,3,4]. DORIS (doppler orbitography and radiopositioning integrated by satellite) is a mature space geodetic technique that is slightly younger than the other three, such as the global navigation satellite system (GNSS), very long baseline interferometry (VLBI), and satellite laser ranging (SLR). Compared to SLR and VLBI, the DORIS system has a uniform global distribution of beacons and high observation accuracy that has advantages in space geodetic research and satellite orbit computation, such as precise orbit determination of Low Earth Orbit (LEO) satellites, calculation of Earth’s rotation parameters, geocentric movement research, establishment of the International Terrestrial Reference Frame (ITRF), vertical crustal movements, etc. [5,6]. Nowadays, this system can determine the orbits of the LEO satellites at a precision level of 1–2 cm, and has been applied in the establishment of ITRF2005, ITRF2008, and ITRF2014 [7,8,9,10,11,12]. Gambis [5] analyzed the short-term PM series calculated by DORIS and found that differences in PM solved by DORIS and that by IERS C04 were 1.13 mas and 0.69 mas, in the X and Y directions, respectively. Lian et al. [13] evaluated the accuracy of the Earth’s rotation parameters solved by DORIS, SLR, VLBI, and GPS, and compared the resolution derived from the four geodetic techniques. Except for the above two studies, there are few other studies that involve PM derived from DORIS.

Different methods and means were investigated and applied for time-series analysis, such as Fourier fast transform (FFT), singular spectrum analysis (SSA), wavelets, etc. FFT is a computational tool that facilitates signal analysis, such as power spectrum analysis and filter simulation, using digital computers. It is a method for efficiently computing the discrete Fourier transform (DFT) of a series of data samples. The efficiency of this method is such that solutions to many problems can now be obtained substantially more economically than in the past. This is the reason for the very great current interest in this technique [14]. The SSA method can effectively identify and display the trend terms and periodic signal of a time-series, allowing the precise separation and reconstruction of its principal components. Therefore, this method has been used in oceanography [15], climatology [16], geodesy and geophysics [17,18,19,20,21,22], and in other fields involving component analyses of time-series. Rangelova et al. [19] extracted the periodic signals from the gravity recovery and climate experiment (GRACE) gravity field solutions by adopting multi-channel singular spectrum analysis (MSSA). Khelifa et al. [20] studied the time-series of DORIS station coordinates by applying the SSA method; the test results showed significant nonlinear movements of several millimeters and were presented in the form of the first reconstructed components (RCs). Xiao et al. [21] extracted the periodic terms for simulated satellite clock errors, using SSA and Fourier band-pass filtering (FBPF) methods. Zhang [22] evaluated the trend and seasonal components extracted from GNSS coordinate time-series by employing the improved SSA method. Shen et al. [23] investigated and predicted the Earth orientation parameter (EOP) 08 C04 PM time-series by adopting an autoregressive moving average model and the SSA method and testified that SSA is an effective method for time-series analysis. The wavelet method is another effective method for analyzing time-series, although tests have proved that this method cannot exactly distinguish the periods of annual wobble and Chandler wobble [24].

At present, there are very few studies on PM based on DORIS, so it is necessary to undertake detailed research to investigate the performance of this time-series. This study focused on the analysis of PM time-series, based on the DORIS space geodetic technique using, FFT and SSA methods. It employed 25-year data from January 1993 to January 2018, compared them with EOP 14 C04 to find the difference between them, and then extracted the trend terms, seasonal signal, and noise by singular value decomposition (SVD). More importantly, the components were reconstructed, and the periods of the main RCs were investigated fully.

This paper was organized as follows. In Section 2, the mathematical models and strategies were described thoroughly. In Section 3, PM time-series derived from DORIS were analyzed and compared with those from EOP 14 C04. Additionally, spectrum analysis was carried out using FFT, and the series were decomposed and reconstructed using the SSA method. The full results are discussed in Section 4, and finally the conclusions are drawn out in Section 5.

## 2. Mathematical Models

### 2.1. FFT

FFT is an algorithm that makes possible the DFT computation of a time-series more rapidly than using the other algorithms available, and it can substantially reduce round-off errors associated with these computations [14]. To obtain a steady PM time sequence, a difference method was applied to the original one.

x(n) is the PM time-series after applying the difference method, and its sample number is N. Its DFT can be defined by:

(1) $$F(k)=\sum_{n=0}^{N-1}x(n)W_{N}^{nk}$$

where F(k) is the k−th coefficient of the DFT, and $W_{N}=e^{-i\frac{2\pi }{N}}$, $i=\sqrt{-1}$.

The calculation quantity of DFT was proportional to $N^{2}$; therefore, to reduce the calculation load of DFT for a long time-series, DFT was decomposed into the sum of several short time-series. For the periodicity, symmetry, and reducibility of $W_{N}^{nk}$, DFT could be given as:

(2) $$\begin{array}{ll} F(k) & =DFT[x(n)]\\ & =DFT[x(2u)]+DFT[x(2u+1)]\\ & =\sum_{u=0}^{N/2-1}x_{1}(u)W_{N/2}^{uk}+W_{N}\sum_{u=0}^{N/2-1}x_{2}(u)W_{N/2}^{uk} \end{array}$$

where $\{\begin{array}{l} x_{1}(u)=x(2u)\\ x_{2}(u)=x(2u+1) \end{array}$, $\{\begin{array}{l} W_{N}^{-nk}=W_{N}^{(N-n)k}=W_{N}^{n(N-k)}\\ W_{N}^{\left(k+N/2\right)}=-W_{N}^{k}\\ W_{N}^{N/2}=-1\\ W_{N}^{0}=1 \end{array}$.

In this work, the FFT algorithm based on time extraction is adopted.

### 2.2. SSA

The SSA method could be used to extract significant components from time-series, such as trends, periodic signals, and noise [20,25,26,27]. The method was based on computation of the eigenvalues and eigenvectors of a covariance matrix C formed from the time-series {x(n),t=1,…,N}, and the reconstruction of this time-series, based on a number of selected eigenvectors associated with the significant eigenvalues of the covariance matrix. The trajectory matrix X could be given as:

(3) $$X=\left[\begin{array}{cccc} x_{1} & x_{2} & \cdots & x_{N-M+1}\\ x_{2} & x_{3} & \cdots & x_{N-M+2}\\ \vdots & \vdots & \ddots & \vdots \\ x_{M} & x_{M+1} & \cdots & x_{N} \end{array}\right]$$

where 1<M<N−L+1.

Having formed the trajectory matrix, the SVD was applied to retrieve its principal components (PCs).

In Formula (3), all elements on the opposite lines were equal. The methods for finding the covariance matrix of a trajectory matrix mainly included the BK (Broomhead and King) and VG (Vautard and Ghil) methods [25,28]. The covariance matrix obtained by the VG method could weaken the noise interference, so the VG method was generally applied to construct the covariance matrix [21]. The covariance matrix could be written as:

(4) $$C_{VG}=\left[\begin{array}{cccc} c\left(0\right) & c\left(1\right) & \cdots & c\left(M-1\right)\\ c\left(1\right) & c\left(0\right) & \cdots & c\left(M-2\right)\\ \vdots & \vdots & \ddots & \vdots \\ c\left(M-1\right) & c\left(M-2\right) & \cdots & c\left(0\right) \end{array}\right]$$

where $c(j)=\frac{1}{N-j}\sum_{i=1}^{N-j}x_{i}x_{i+j}$ and j=0,1,…,M−1.

Based on $C_{VG}$, the eigenvalue $\lambda_{k}$ and eigenvectors $E_{j,k}$ could be computed. The eigenvalues could be sorted on the basis of their sizes as $\lambda_{1}\geq \lambda_{2}\geq \cdots \geq \lambda_{M}$, and the corresponding eigenvectors were $E_{1},E_{2},\ldots E_{M}$.

If the eigenvector corresponding to eigenvalue $\lambda_{k}$ was $E_{j,k}$, the time-series could be constructed by:

(5) $$a_{i,k}=\sum_{j=1}^{M}x_{i+j}E_{j,k}$$

where $a_{i,k}$ is the k−th PC.

According to Formula (5),

(6) $$R_{i,k}=\{\begin{array}{ll} \frac{1}{i}\sum_{j=1}^{i}a_{i-j+1,k}E_{j,k}, & 1\leq i\leq M-1\\ \frac{1}{M}\sum_{j=1}^{i}a_{i-j+1,k}E_{j,k}, & M\leq i\leq N-M+1\\ \frac{1}{N-i+1}\sum_{j=i-N+M}^{M}a_{i-j+1,k}E_{j,k}, & N-M+2\leq i\leq N \end{array}.\;$$

The series $R_{i,k}$ of length N were called the RCs, and they kept the phase properties of the original time-series.

## 3. PM Analysis Using FFT and SSA

For this work, we used the daily PM time-series derived from the DORIS weekly solutions of coordinates for the time-period of January 1993 to January 2018 (ina18wd01), available on the Internet (ftp://cddis.gsfc.nasa.gov/pub/doris/products/). These data were calculated weekly at the Russian Academy of Sciences Institute of Astronomy (INASAN) analysis center using DORIS data and GIPSY/OASIS II software, which was developed by the Jet Propulsion Laboratory (JPL) [29] and was significantly expanded for DORIS applications [30] by a joint cooperation between the Institut Géographique National (IGN) and JPL. This package was also adapted by the IGN for the DORIS data analysis [31].

### 3.1. DORIS PM Analysis and Comparison with EOP 14 C04

**Figure 1:**
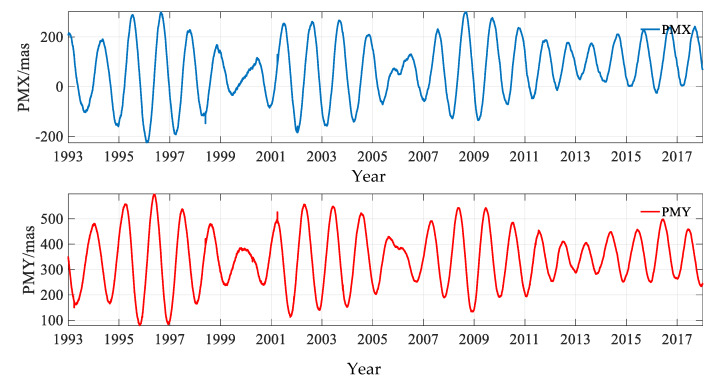
PM time-series solved from the DORIS data for the period of January 1993 to January 2018.

**Figure 2:**
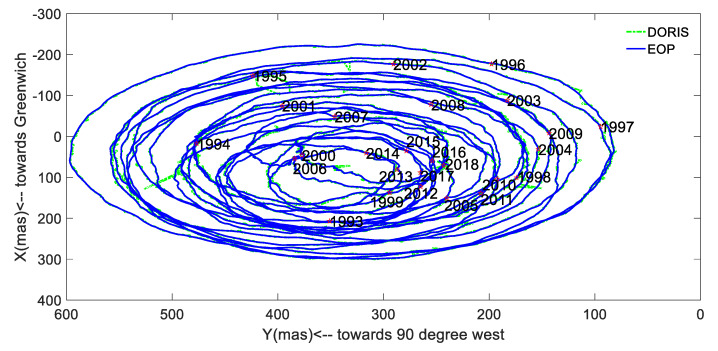
‘Polhody’ over the period of January 1993 to January 2018, obtained from the DORIS observations and EOP 14 C04.

In order to display the variation of the PM amplitude in the X (PMX) and Y (PMY) directions, the time-series is plotted in Figure 1. To show the intersections named ‘polhody’ between the Earth’s pole of rotation and the Earth’s surface, the time-series is shown in Figure 2.

Figure 1 displays the values of PM in the X and Y directions, and the maximum values were 300.515 mas and 596.492 mas, respectively. Figure 2 shows that the ‘polhody’ from January 1993 to January 2018 solved from the DORIS observations had a good agreement with the results published by IERS (https://www.iers.org/IERS/EN/DataProducts/EarthOrientationData/eop.html), using EOP 14 C04 data derived from multiple space geodetic techniques.

**Figure 3:**
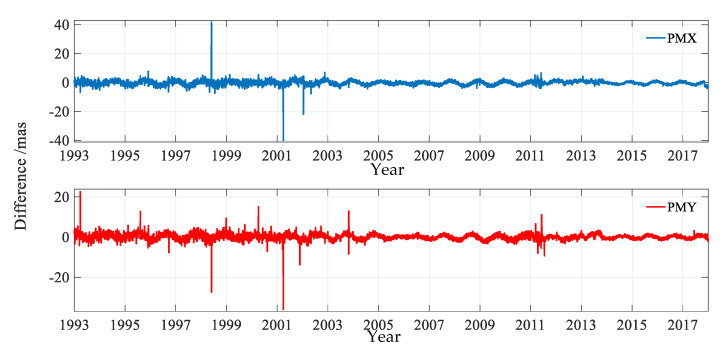
Differences in PM derived from DORIS and EOP 14 C04 over the period of January 1993 to January 2018.

In order to compare the PMs solved from DORIS and EOP 14 C04, the differences in the two components were calculated as shown in Figure 3; the statistical information is provided in Table 1.

Figure 3 and Table 1 reinforce that there was good agreement of the PM derived from DORIS and EOP 14 C04. Table 1 shows that the maximum differences in the X and Y directions were 41.779 mas and 22.557 mas, and the root mean squares (RMSs) in the two directions were 1.594 mas and 1.465 mas, respectively. Figure 3 shows that there were some small periodic signals. The periodicity of the difference was related to many aspects. The parameters of EOP 14 C04 were together solved by VLBI, SLR, GNS, and DORIS, therefore, the scales and solution strategies were different. Even for DORIS, there were mainly six analysis centers to provide data. The analysis software, models, algorithms, and strategies of these six analysis centers were different too, such as the gravity field model, the satellites used, the satellite cutoff angles, and the phase center (corrected or not), etc. All these factors led to the systematic periodic difference in the whole time-period.

Figure 3 also displays some significant peaks. There was a peak on 20 March 1993; the main cause was unknown as there was no record of a solar storm, earthquake, geomagnetic storm, data gap, and so on. Another peak was on 4 December 1995, and this could be related to the fact that the DORIS data were valid for SPOT-3 and T/P satellites over the time-span 3 December to 5 December 1995. There were 4 peaks of the differences in the X and Y components over the time-span 30 May to 2 June 1998, respectively, which were mainly due to the erroneous values of the center of mass correction of the SPOT-4 satellite, during this time-period. There was a peak in 7 April 2000, and the main cause was that, over the time-period of 2–6 April 2000, there were three DORIS DOPPLER instrument nominal modes with median frequency bandwidth pre-positioning, which led to less available data and two single event upsets for the TOPEX/POSEIDON. Additionally, there was one orbit maintenance maneuver for the SPOT-2 satellite. There was a peak on 24 November 2001, and this was mainly due to two orbit maintenance maneuvers for SPOT-2 and SPOT-4. Additionally, two beacon stations ended work and two began work, and an earthquake with magnitude 6.3 on the FUTB station might have also contributed to the peak. There was a peak on 31 March 2001, which mainly relates to the data gap of SPOT-2 and because AMSB ended work and AMTB started work. There was a peak on 14 January 2002, and this was mainly due to the data format changes for the SPOT-2 and SPOT-4 satellites.

It can be observed from Figure 3 that the difference in polar motion was large and unstable from 1993 to April, 2002. This effect could be explained by the fact that only the data from the SPOT-2, SPOT-3, SPOT-4, and TOPEX/POSEIDON were applied, and the receivers on board were first generation, which could only track 1 beacon at a time. After April 2002, the DORIS data from the SPOT-5 and ENVISAT satellites were added, and the receivers loaded on both satellites were second generation receivers that could track 2 beacons at a time. The significant precision improvement of the X and Y pole components after April 2002 was related to the increase of the satellite number from 3 to 5. Every time a new satellite was added, the quality of the PM parameter series got better [32]. After May 2004, the difference became small and stable; the main reasons were the increasing number of satellites and beacon stations, and the new generation of receiver on board also contributed to the high accuracy and stability of the polar motion parameters.

It can also be seen from Figure 3 that the difference values changed greatly from March to May in 2011, and this fact mainly related to 8 interruptions of the DORIS data, including RILB beacon and antenna failures, NOWB beacon replacement, CADB beacon interrupted due to works on site, the invalid data of CRYOSAT-2 and JASON-2 due to the new DORIS data format, and the instrument failure and invalid data of ENVISAT over the time-period of 21–23 May.

### 3.2. FFT Analysis of the Time-Series

**Figure 4:**
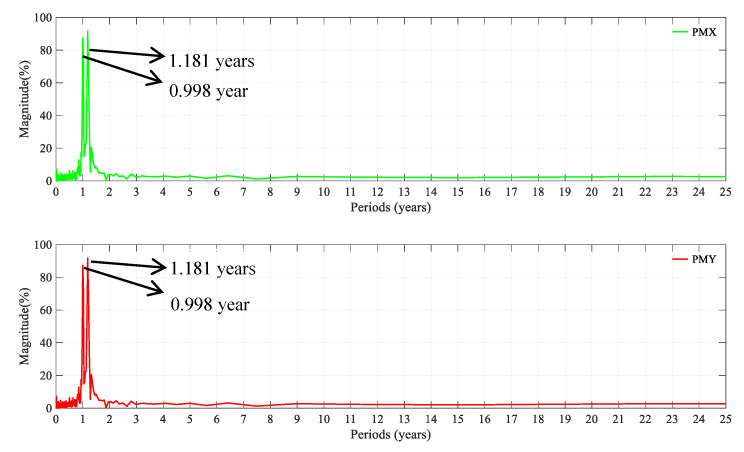
PMX and PMY periodograms using FFT.

To present and validate the period of PM, the time-series in the two components were varied to get steady time-series in two directions, and then FFT was performed for both time-series; finally, the power spectrums are shown in Figure 4.

Figure 4 reveals two important peaks in both PMX and PMY, which indicate that these two peaks were responding to the 0.998-year annual signal and 1.181-year signal, respectively. The 1.181-year term represents the period of Chandler wobble, which is a free oscillation, and the annual one is the annual wobble, which is a natural oscillation. In both directions, the magnitude of 1.181 years was more powerful than that of 0.998 years, which suggests that the Chandler wobble had a more severe oscillation than the annual one.

### 3.3. SSA of the PM and Analysis of the Main RCs

**Figure 5:**
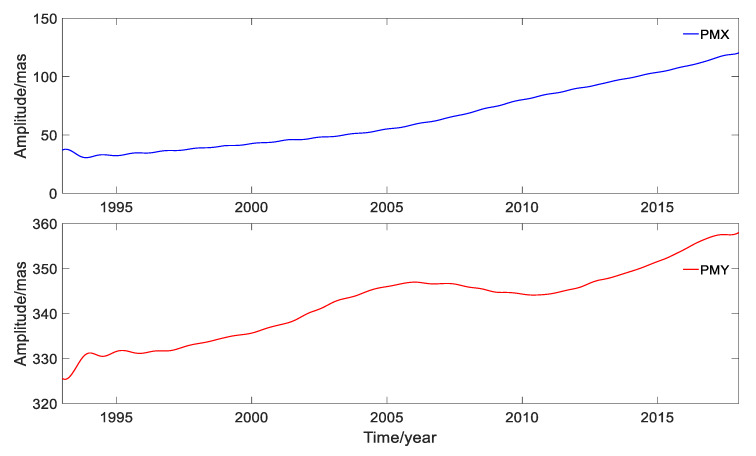
PM trend in the X and Y components extracted using SSA.

**Figure 6:**
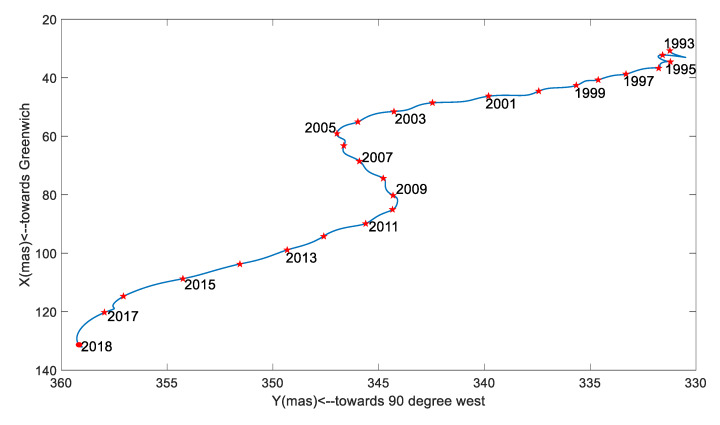
PM trend variation over the period of January 1993 to January 2018.

**Figure 7:**
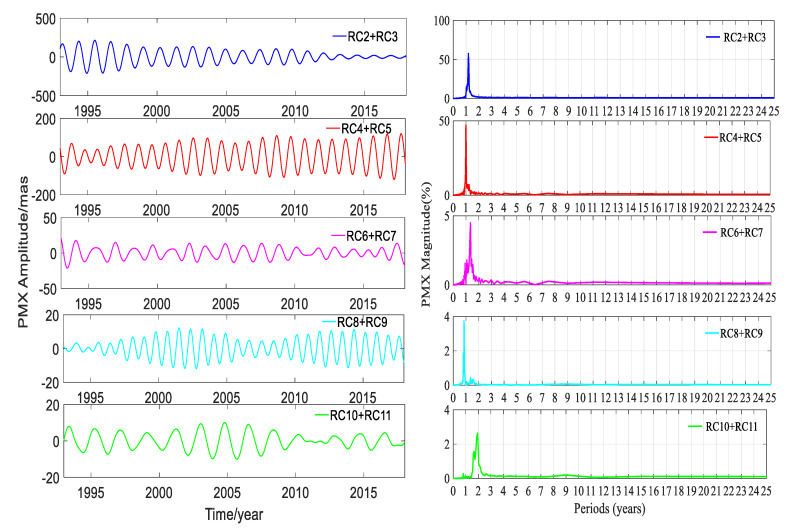
Amplitudes and periodograms of the main reconstructed components (RCs) of PMX solved by DORIS.

**Figure 8:**
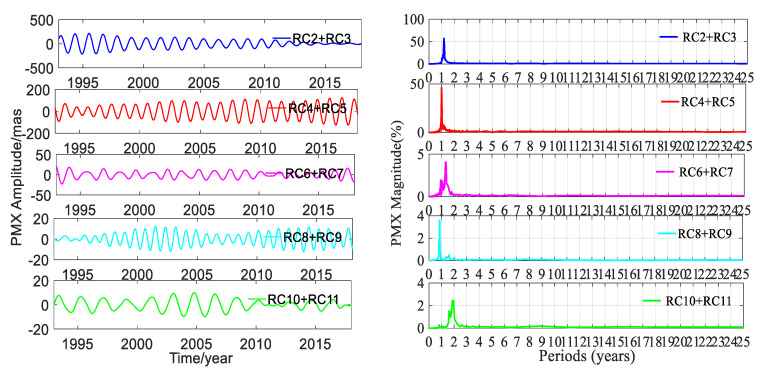
Amplitudes and periodograms of the main RCs of PMX from EOP 14 C04.

**Figure 9:**
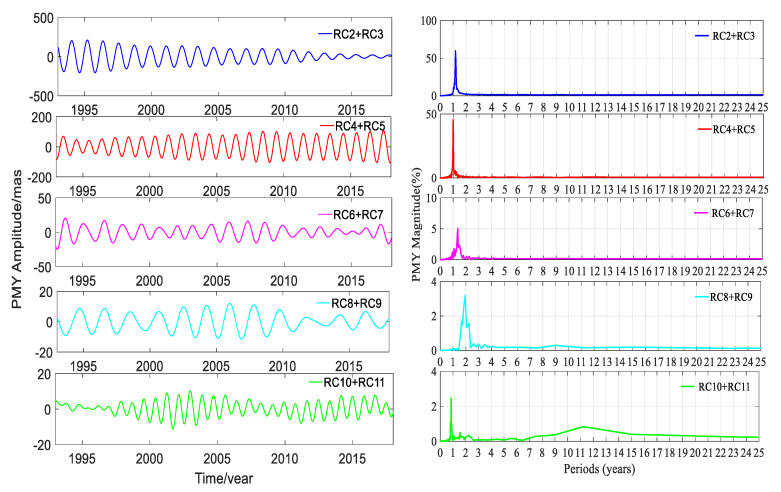
Amplitudes and periodograms of the main RCs of PMY from DORIS.

**Figure 10:**
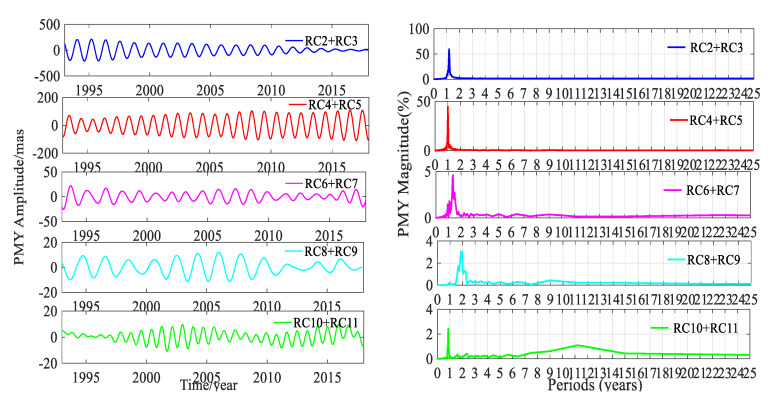
Amplitudes and periodograms of the main RCs of PMY from EOP 14 C04.

SSA could easily extract the trend terms; besides this, the other main ability of SSA was to automatically and easily detect the dominant periodic signals and reconstruct them. A pair of equality eigenvalues was associated with a dominant seasonal signal. The time window length was chosen as 6 years, and the time-period was 25 years from January 1993 to January 2018. To gain insight into the trend terms, the seasonal signals and the gross errors, the SSA method was applied to detect and extract these terms in this work. The trend terms in the two directions are shown in Figure 5. According to the trend signals, the variation in the 25-year PM trend is displayed in Figure 6. In order to make a full assessment of the periods of seasonal signals, the RCs were formed; periodograms of the RCs in the X and Y directions derived from the DORIS and EOP 14 C04 are shown in Figure 7, Figure 8, Figure 9 and Figure 10, respectively, and the statistics are summarized in Table 2.

Figure 5 shows that the PMX trend exhibited an approximately linear change, while the PMY trend exhibited a significantly nonlinear change. The rate in X was about 3.3308 mas/year; in Y it was about 1.2965 mas/year. Generally, the PM trend rate was about 3.5742 mas/year, in the southwest direction.

Figure 6, however, shows that the path of polar movement relative to the Earth’s crust was not a straight line. It was significant that from January 1993 to November 2004, the trend trajectory was almost linear with a rate of 1.8907 mas/year, then shifted suddenly in December 2004, and from December 2004 to February 2010 the movement with a rate of 0.6336 mas/year shifted by about 90 degrees, relative to the path from January 1993 to December 2004. Similarly, from March 2010, there was another sudden curve in the PM trajectory, with a rate of 3.0157 mas/year, and from March 2010 to January 2018, the trajectory of the move was almost parallel to the one from January 1993 to December 2004. Figure 6 also shows that the directions of north pole movement were different during the three time-periods. From January 1993 to December 2004, the north pole moved about 83.0248 degrees southwest in a longitudinal direction with respect to the crust; from December 2004 to March 2010, the movement was about 10.8872 degrees southeast, and from March 2010 to January 2018, it was about 74.1812 degrees southwest. The tendency of the polar motion separated using SSA showed a good agreement with the result obtained from reference [33]. To a certain extent, five large earthquakes bigger than 8.5 magnitudes, over 2004 to 2010, contributed to these sudden dramatic changes [34], and other unknown reasons had direct relation to this phenomenon [32].

Figure 7, Figure 8, Figure 9 and Figure 10 and Table 2 show that reconstructed polar motion signals of every corresponding component solved using DORIS and EOP 14 C04 by SSA had the same periods, and the magnitudes were a little different. As displayed in Figure 7, Figure 8, Figure 9 and Figure 10 and Table 2, the RCs and periodograms had similar features for the X and Y components. RC2 and RC3 were a pair of main components. The periodograms indicated that this pair of signals had the same 1.181-year period in both directions and their magnitudes were all over 57.1%. These components corresponded to the Chandler wobble. The important information detected in Figure 7, Figure 8, Figure 9 and Figure 10 was that the amplitudes of RC2 + RC3 in both directions had the same significant decay phenomena, and the oscillation almost stopped at the end of the period. The main reason was that the Chandler wobble was excited mainly by the atmospheric process (wind and surface pressure variations), elastic deformation of the Earth’s mantle, and mass movement in the oceans and the liquid outer core of the Earth, due to the variable centrifugal force generated by the Earth’s rotation. This oscillation was a kind of damping motion, which theoretically led to the amplitude gradually decaying and finally stopping if there were no excitation sources. Studies have shown that the amplitude of this swing is increased again, after a period of attenuation [35]. In addition to the damping mechanism, the Chandler wobble also has a random excitation mechanism. During a weak excitation period, the vibration amplitude is attenuated or even terminated; during a strong excitation period, the oscillation amplitude is increased. The excitation mechanism of the oscillation has three main aspects—the impact of earthquakes, the impact of the atmosphere and oceans, and changes in water storage [35].

Similarly, Figure 7, Figure 8, Figure 9 and Figure 10 and Table 2 also indicate that RC4 and RC5 were a pair in the X and Y directions and had the same 0.998-year period. At the same time, Table 2 also showed that the magnitudes of RC4 + RC5 were all larger than 42.6%, which indicated that RC4 and RC5 were another pair of main components. These components correspond to the annual wobble which was a seasonal signal. The annual pole movement was due to seasonal atmospheric, oceanic, and groundwater distribution processes, and the main cause was the annual inertial variation that was accompanied by seasonal redistribution of the mass of the atmosphere. The redistribution of mass and the global response of the oceans to annual changes in atmospheric pressure, could also stimulate the annual pole shift by changing the inertia product. In addition, the annual PM could also be stimulated by a change in the mass distribution of the annual changes of water storage, including snow and ice [35].

The above analysis confirmed that the SSA method could significantly separate the main signals, and reinforced that the PM had a Chandler wobble and an annual wobble. Similarly, RC6 and RC7 were a pair of components for PMX and PMY; they had the same period of 1.360 years, and both had a magnitude of about 5.0%. These oscillation terms derived from DORIS and EOP 14 C04 had the same periods as the results calculated by Shen et al. [23], using EOP 08 C04 PM series.

For RC8 + RC9 of PMX, there was a noticeable peak corresponding to 0.847 years on the periodogram in Figure 7 and Figure 8, as listed in Table 2. While for the same pair for PMY, the outstanding peak value on the periodogram was 1.952 years, which was same as that for RC10 + RC11 for PMX. Additionally, there was another signal with an 8.978-year period which agreed with the period of about 9.0 years given by Schuh et al. [36]. For RC10 + RC11 in the Y direction, the periodograms in Figure 9 and Figure 10 show two remarkable magnitude peaks; the corresponding statistics are given in Table 2. The above information suggests that this pair of terms contained two period signals, 0.863 and 11.22 years, respectively. The 0.863-year period was similar to the 0.847 years of RC8 + RC9 in PMX and had a good agreement with the results obtained by Guo and Han [24], based on SLR data, and the 11.22-year period had a good agreement with the result of Schuh et al. [36]. The analyses indicated that there were similar period signals in PMX and PMY; however, these similar period signals had different contributions from their own original series, which could be reinforced by their magnitudes.

From the above analysis, it could be concluded that the trend terms and main seasonal signals could be clearly separated by the SSA method. In addition, long-term signals such as the approximately 9- and 11-year period signals were also extracted, which were in good agreement with the results of Schuh et al. [36]. However, their magnitudes were very small. The main reason might be that the DORIS PM time-series was not long enough. Additionally, the above analysis indicated that the Chandler wobble and the annual wobble were the main periodic terms of motion, which was in good agreement with the results of the FFT spectrum analysis in this work.

Table 2 also displays that the amplitudes of the signals in DORIS were very close to those of EOP 14 C04, except RC4 + RC5 in the X and Y directions. The amplitudes of RC2 + RC3, RC4 + RC5, RC6 + RC7, and RC8 + RC9 from DORIS were smaller than those of EOP 14 C04 in the X and Y components. On the contrary, the amplitudes of RC10 + RC11 from DORIS were larger than those of EOP 14 C04 in both components, but the differences of these amplitudes were very small and they were 0.076 and 0.685 mas in the X and Y components, respectively. As for the other pairs of RCs, the differences of amplitudes were all below 1.175 mas except RC4 + RC5 in both directions. The differences of amplitudes for RC4 + RC5 were 4.460 and 2.538 mas between DORIS and EOP 14 C04 in the X and Y components, respectively, and they were larger than those of other RCs. The differences of amplitudes showed a relation with the many factors. First, the polar motion parameters of EOP 14 CO4 were solved with four combined space geodetic observations, such as SLR, VLBI, GNSS, and DORIS, and these four space geodetic techniques had different scales and tracking station networks. Second, most of the data applied to solve the polar motion parameters were from different satellites. Third, the software, models, algorithms, and strategies used to solve the PM parameters for DORIS and EOP 14 C04 were different. RC4 + RC5 was the annual wobble. The significant difference of amplitudes was from RC4 + RC5, which was the annual wobble signal, and the reasons for this interesting fact are unknown and needs to be further investigated.

**Figure 11:**
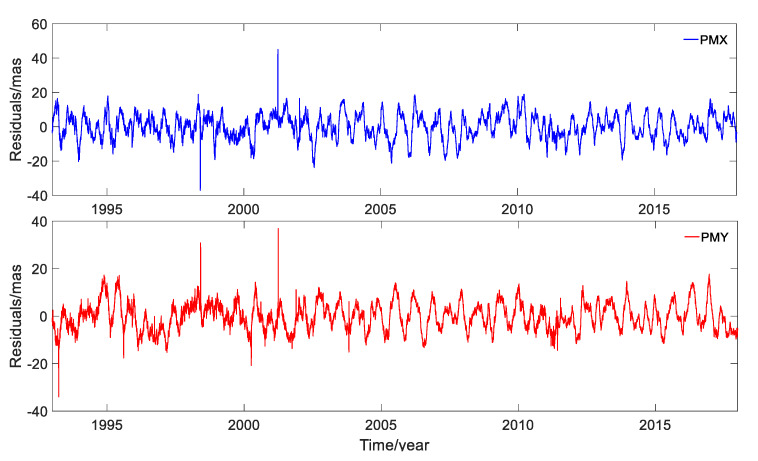
Residuals of PMX and PMY after removing the first 11 RCs.

The first 11 RCs in the X and Y directions accounted for 99.49% and 99.93% of the corresponding original time-series solved by DORIS, respectively. Determination of the gross noise by the SSA approach was based on the removal of the trend and various seasonal components from the original time-series; the residual terms after deducting the first 11 RCs are shown in Figure 11 and the statistics are listed in Table 3.

It can be observed from Figure 11 and Table 3 that the RMSs of residual components were 7.050 mas and 6.007 mas, respectively, and they accounted for 0.51% and 0.07% of the RCs for PMX and PMY, respectively. In general, the residuals with such small contribution could be regarded as noise. However, as can be seen in Figure 11, there still seemed to be some periodic variations clearly visible. According to the analysis, we could not conclude that the residuals were gross noise.

**Figure 12:**
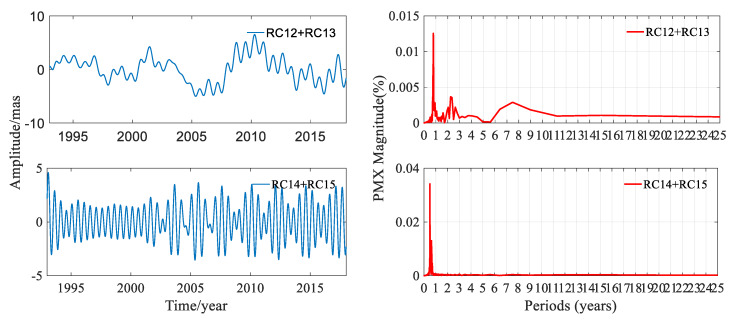
Amplitudes and periodograms of RC12 + RC13 and RC14 + RC15 of PMX from DORIS.

**Figure 13:**
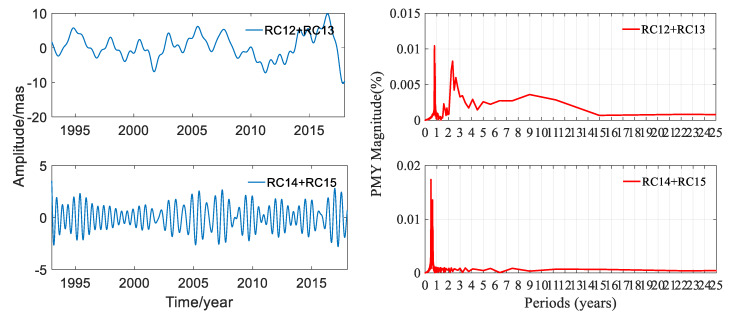
Amplitudes and periodograms of RC12 + RC13 and RC14 + RC15 of PMY from DORIS.

In order to investigate the components of polar motion in depth, the RC12 + RC13 and RC14 + RC15 were studied. RC12 + RC13 was not a stationary time-series. In order to make a better periodic analysis of this pair of RCs, first differences between consecutive data were computed and then FFT was performed. The amplitudes and the periodograms of PMX and PMY of these two pairs of RCs are shown in Figure 12 and Figure 13, respectively; the statistics are listed in Table 4.

Figure 12 and Table 4 displayed that the periods of RC12 + RC13 for PMX were 0.788, 2.244, and 7.481 years, and the amplitude was 6.507 mas. The period of RC14 + RC15 was 0.499 year, and the amplitude was 4.592 mas in the X direction. It can be seen from Figure 13 and Table 4 that the periods of RC12 + RC13 for PMY were 0.802, 2.363, and 8.978 years, and the amplitude was 9.884 mas. The period of RC14 + RC15 for PMY was 0.499 year, and the amplitude was 3.638 mas. These signals were small compared to the full signal, while the periods of RC12 + RC13 and RC14 + RC15 were significant. It should be noted that the result indicated that RC14 + RC15 was a semi-annual signal.

**Figure 14:**
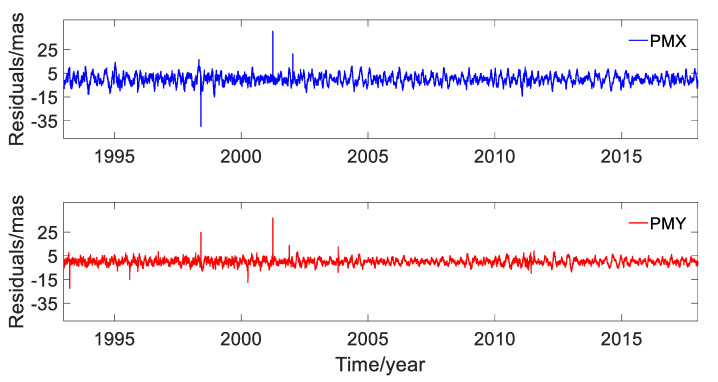
Residuals of PMX and PMY after removing the first 15 RCs.

The residuals of PMX and PMY after removing the first 15 RCs are plotted in Figure 14. The RMS of the residuals were 3.858 and 2.387 mas in the X and Y directions, respectively.

Figure 14 displays that the residual time-series had some periodic variations after removing the first 15 RCs, and it was hard to judge if this was true. Compared to Figure 11, the residuals were smaller and thus were regarded as noises in this work. The residuals will make for further intensive investigation in future work.

## 4. Discussion

Today, there are various geodetic techniques with the ability to determine PM, such as SLR, VLBI, and GNSS, and more recently DORIS was introduced in IERS activities from 1995 [5]. DORIS, as a space geodetic technique, can determine ground beacon positions and was introduced in the ITRF, with an accuracy of 10 mm. So far, the accuracy of PM derived from DORIS compared to other individual series can reach 1–3 mas [5].

In this work, to assess the performance of PM based on the DORIS space geodetic technique, the PM series solved by DORIS and EOP 14 C04, which is based on multiple space geodetic techniques, were compared and evaluated for the time-period of January 1993 to January 2018. To obtain the trend terms, seasonal signals and noise of this PM time-series, the SSA method was applied to retrieve the PCs and reconstruct the components; the trend terms are discussed in detail. In order to analyze the periodic characters of the time sequence and main RCs, the FFT method was adopted to obtain their spectrum information, and the periodic signals and their corresponding geophysical factors were analyzed in detail.

Previous studies have described the PM solved by SLR, VLBI, GPS, and multiple techniques [5,22,36]. These studies neither determined the DORIS-based PM nor separately assessed the performance of DORIS. Gambis [5] carried out a comparison of the different PM, based on DORIS solved by different independent analysis centers, and performed spectral analysis only using FFT, but did not carry out a full analysis of the main components of PM derived from about 13 years of DORIS data. Shen et al. [23] used the Toeplitz method to form a covariance matrix for the EOP 08 C04 data and finally separated the trend terms, seasonal signals, and the noise of the PM, based on multiple techniques, but the study failed to detect the damping phenomena of the Chandler wobble. Schuh et al. [36] applied the FFT method to analyze the PM periodograms for the C01 series published by IERS; however, this study did not separately analyze the period spectrums of PMX and PMY.

DORIS, as a recently developed space geodetic technique, has great potential for orbit determination, ITRF construction, navigation, and so on, and most studies adopted this technique to determine satellite orbits. Many research studies have analyzed the accuracy of PM based on SLR, GNSS, or multiple techniques. This paper not only determined the difference between PM derived from DORIS and EOP 14 C04, but also showed a full analysis of the main components of PM.

PM plays an important role in geophysical research, determination of the LEO orbit, satellite navigation, and so on. The study provides a theoretical and methodological reference for the analysis of PM and its further prediction that will help in the analysis of the structure and composition of the Earth.

## 5. Conclusions

The main purpose of this article was to apply FFT and SSA methods to analyze the 25-year PM series solved from the DORIS space geodetic technique; the analysis allows one to fully investigate the trend terms, seasonal signals, and noise for PM derived from DORIS. In addition, this study also focused on proving the performance of DORIS and promoting more fields of application for this newly developed space geodetic technique.

In order to find out the periodic signals of PM, the FFT and SSA methods were used, and the Chandler wobble with a 1.181-year period, the annual wobble with a 0.998-year period, and the semi-annual signal with a 0.499 year were successfully detected. To prove the perfect performance of DORIS for solving PM, DORIS PM was compared with that derived from the EOP 14 C04 data, and the tests indicated that they agreed well with each other. In addition, the causes of difference peaks were provided; the RMSs in X and Y directions were 1.594 and 1.465 mas, respectively, which suggested that the DORIS PM was reliable. To make a detailed assessment of the time-series, the SSA approach was adopted to decompose the series. The components were reconstructed using eigenvalues and eigenvectors, then the periods of every pair of main RCs were detected using the FFT method and were analyzed in detail. Tests proved that the trend terms, seasonal signals, and noise were extracted successfully.

To assess the nonlinear trend terms and seasonal signals, a full analysis was performed. The nonlinear trend terms indicated that the poles moved toward the southwest and that the directions for the three different time-periods were all different. From January 1993 to November 2004, the trend trajectory was almost linear; from December 2004 to February 2010, there was a sudden shift in direction, and from March 2010 to January 2018, the direction was almost parallel to that for January 1993 to November 2004. The above phenomena showed a close relation with five large earthquakes during 2004–2010 [34]. According to the first 15 RCs and the corresponding periodograms, it was concluded that the SSA could successfully extract the trend and the seasonal terms. In addition, the results also suggested that the amplitudes of Chandler terms in the studied time-period had a distinct attenuation trend, which enforced the idea that the oscillation was a damping motion. The noise determined by the SSA method was based on the removal of the trend and various seasonal components from the original PM time-series, and the RMSs of the noise in the X and Y directions were 3.858 mas and 2.387 mas, respectively. Therefore, the conclusion could be drawn that the SSA method had the remarkable ability to separate noise from the original series and to successfully remove it during the process of component reconstruction.

However, for further long-term trend analysis of DORIS PM, greater efforts should be put into researching the mechanism of PM derived from this space geodetic technique, which would provide further deep comprehension of the Earth’s structure, inner material movement, water storage changes, ice melting, and so on from the viewpoint of this newly developed technique. In addition, a PM period longer than 10 years was detected, while the spectrum of this component was not outstanding, as compared to the other main components. Therefore, further study would lengthen the time-period of the time-series, solved by the DORIS data. All these research studies would provide an important theoretical basis for the prediction of PM and the study of geo-hazards, tectonics, Earth structure and composition, and so on.

## Figures and Tables

**Table 1:** Statistics of the differences in PM derived from DORIS and EOP 14 C04 (mas).

Direction	Max	Min	Mean	STD	RMS
X	41.779	−40.014	1.087	1.584	1.594
Y	22.557	−35.919	1.026	1.460	1.465

**Table 2:** Statistics of the main components of PMX and PMY.

		PMX					PMY						
	RC	RC2 + RC3	RC4 + RC5	RC6 + RC7	RC8 + RC9	RC10 + RC11	RC2 + RC3	RC4 + RC5	RC6 + RC7	RC8 + RC9		RC10 + RC11	
DORIS	Period (years)	1.181	0.998	1.360	0.847	1.952	1.181	0.998	1.360	1.952	8.978	0.863	11.220
	Magnitude (%)	57.550	47.050	4.497	3.742	2.634	59.590	45.550	5.051	3.194	0.294	2.475	0.835
	Amplitude (mas)	213.311	119.924	20.880	12.117	10.063	209.449	106.554	20.576	12.133		10.337	
EOP 14 C04	Period (years)	1.181	0.998	1.360	0.847	1.952	1.181	0.998	1.360	11.952	8.978	0.863	11.220
	Magnitude (%)	57.100	42.620	6.060	3.599	2.458	59.400	44.750	4.611	3.068	0.440	2.440	1.095
	Amplitude (mas)	213.562	124.384	21.864	12.128	9.987	210.416	109.092	21.751	12.160		9.652	

**Table 3:** Statistics of the residuals of PMX and PMY after removing the first 11 RCs (mas).

PMX (mas)				PMY (mas)			
Max	Min	Mean	RMS	Max	Min	Mean	RMS
45.007	−37.133	0.0804	7.050	36.883	−34.010	−0.129	6.007

**Table 4:** Statistics of two pairs of small components of PMX and PMY.

	PMX		PMY	
RC	RC12 + RC13	RC14 + RC15	RC12 + RC13	RC14 + RC15
Period (years)	0.788, 2.244, 7.481	0.499	0.802, 2.363, 8.978	0.499
Magnitude (%)	1.25	1.304	1.045, 0.830, 0.361	1.740
Amplitude (mas)	6.507	4.592	9.884	3.638

## Abbreviations

The following abbreviations are used in this manuscript:

DFT	Discrete Fourier Transform
DORIS	Doppler Orbitography and Radiopositioning Integrated by Satellite
FBPF	Fourier Band-Pass Filtering
FFT	Fourier Fast Transform
GNSS	Global Navigation Satellite System
GRACE	Gravity Recovery and Climate Experiment
IGN	Institute Geographique National
INA	Institute of Astronomy
INASAN	Russian Academy of Sciences
ITRF	International Terrestrial Reference Frame
JPL	Jet Propulsion Laboratory
LEO	Low Earth Orbit
MSSA	Multi-Channel Singular Spectrum Analysis
PC	Principal Component
PM	Polar Motion
PMX	Polar Motion in X Direction
PMY	Polar Motion in Y Direction
RC	Reconstructed Components
RMS	Root Mean Square
SLR	Satellite Laser Ranging
SSA	Singular Spectrum Analysis
SVD	Singular Value Decomposition
VLBI	Very Long Baseline Interferometry
